# Composite Cryogel with Polyelectrolyte Complexes for Growth Factor Delivery

**DOI:** 10.3390/pharmaceutics11120650

**Published:** 2019-12-04

**Authors:** Bolat Sultankulov, Dmitriy Berillo, Sholpan Kauanova, Sergey Mikhalovsky, Lyuba Mikhalovska, Arman Saparov

**Affiliations:** 1 Department of Chemical Engineering, School of Engineering, Nazarbayev University, Nur-Sultan 010000, Kazakhstan; bsultankulov@nu.edu.kz (B.S.); skashikova@nu.edu.kz (S.K.); 2 Department of Biotechnology, Al-Farabi Kazakh National University, Almaty 050040, Kazakhstan; dmitriychemist@gmail.com; 3 School of Pharmacy and Biomolecular Sciences, University of Brighton, Brighton BN2 4GJ, UK; l.mikhalovska@brighton.ac.uk; 4 ANAMAD Ltd., Falmer, Brighton BN1 9SB, UK; sergeymikhalovsky@gmail.com; 5 Chuiko Institute of Surface Chemistry, Kyiv 01364, Ukraine; 6 School of Medicine, Nazarbayev University, Nur-Sultan 010000, Kazakhstan

**Keywords:** biomaterials, cryogel, drug delivery, growth factors, tissue engineering, bone regeneration

## Abstract

Macroporous scaffolds composed of chitosan (CHI), hydroxyapatite (HA), heparin (Hep), and polyvinyl alcohol (PVA) were prepared with a glutaraldehyde (GA) cross-linker by cryogelation. Addition of PVA to the reaction mixture slowed down the formation of a polyelectrolyte complex (PEC) between CHI and Hep, which allowed more thorough mixing, and resulted in the development of the homogeneous matrix structure. Freezing of the CHI-HA-GA and PVA-Hep-GA mixture led to the formation of a non-stoichiometric PEC between oppositely charged groups of CHI and Hep, which caused further efficient immobilization of bone morphogenic protein 2 (BMP-2) possible due to electrostatic interactions. It was shown that the obtained cryogel matrix released BMP-2 and supported the differentiation of rat bone marrow mesenchymal stem cells (rat BMSCs) into the osteogenic lineage. Rat BMSCs attached to cryogel loaded with BMP-2 and expressed osteocalcin in vitro. Obtained composite cryogel with PEC may have high potential for bone regeneration and tissue engineering applications.

## 1. Introduction

Bone regeneration is one of the most actively researched fields of regenerative medicine, and bone fractures are the most common injuries of all large organs, especially in the aging population. Moreover, bone restoration is also required in congenital bone defects, tumors and infections [1]. Critical size defects require large-scale surgical interventions, and autografting is accepted as the gold standard treatment due to its osteogenic, osteoconductive, and osteoinductive potentials [2]. However, problems such as a shortage of allografts, rejection issues, and associated pain and morbidity from autografts require the development of alternative tissue engineering approaches that combine the principles of engineering and biology to create biomaterials, which are able to mimic or regenerate functionally active tissues [3]. This has resulted in the development of a variety of natural and synthetic polymer-based materials and methods for the production of functionally active biomaterials [4].

Tissue engineering uses engineering approaches and biological principles to recreate or regenerate living tissues [5]. The development of safe and effective biomaterials has a major role in this process. High demand for materials that could be used to regenerate tissues and organs has stimulated research on the development of biomaterials based on synthetic and natural polymers [4,5,6]. During the development of bioactive scaffolds, it is important to mimic the three-dimensional structure of the extracellular matrix (ECM) of the target tissue, which will be able to provide structural support and maintain cell proliferation, adhesion, and differentiation and biodegrade in an appropriate time period to allow replacement with the native ECM [7].

Hydrogels are widely studied in the regeneration of soft tissue due to properties such as injectability, formation of a three-dimensional network, and cell encapsulation capability [8,9]. Despite these advantages, the nanoporous structure of hydrogels limits proper vascularization as well as cell and nutrient migration to some degree, thus decreasing cell viability. Alternately, a hydrogel precursor could be physically or chemically cross-linked in the frozen system where the chemical reaction leads to gelation in the frozen state [10,11,12,13]. Through this controlled freeze-thaw process, a cryogel can be formed with pores of a different size and increased interconnectivity. Cryogels synthesized from natural polymers have a macroporous structure and, due to their good mechanical properties, are favorable scaffold candidates for bone regeneration [12]. Crosslinking of cryogel precursors occurs at a sub-zero temperature, resulting in the formation of water ice crystals and holes within the cross-linked polymer, creating a highly porous and interconnected structure [10,14].

Due to the biocompatibility and biodegradability of chitosan (CHI), natural CHI scaffolds are promising tools for bone regeneration application [15,16]. Structurally, the bone is composed primarily of a collagen extracellular matrix, which consists of 90% type I collagen, incorporated with hydroxyapatite (HA) (Ca_10_(PO_4_)_6_(OH)_2_) crystals. On average, compact bone is composed of 70% calcium salts and 30% matrix [17,18]. Mineralized scaffolds have been shown to support osteogenic activity and overall bone formation [19]. The presence of HA assists in the imitation of natural bone extracellular matrix (ECM), providing a native chemical and physical structure for new bone to form. Therefore, it is beneficial to increase the rate of HA formation in bone tissue healing [20]. In addition, HA is known to be biocompatible and osteoconductive, and is currently used in many orthopedic applications, including bone fillings and implant coatings [21]. Another main issue that should be considered during the design of a scaffold suitable for bone regeneration is the ability of the scaffold to support vasculogenesis, which could be done by either immobilization of growth factors or by inclusion of substances with an intrinsic ability to bind growth factors [22].

**Figure 1:**
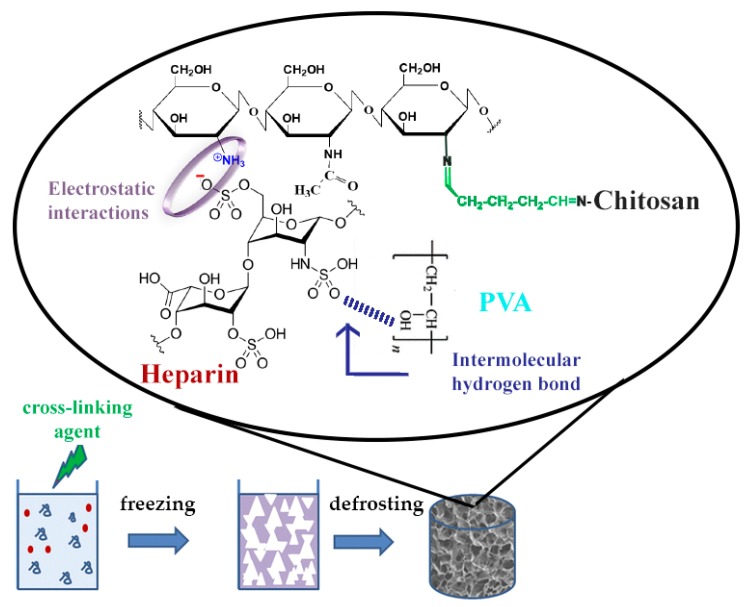
Schematic diagram of polyelectrolyte complex (PEC)-based chitosan (CHI)-polyvinyl alcohol (PVA)-heparin (Hep)-glutaraldehyde (GA) cryogel synthesis showing electrostatic interactions between CHI and Hep and an intermolecular hydrogen bond between PVA and Hep.

The current study is focused on a one-step preparation of heparin (Hep)-containing composite cryogel. Usually, protonated amino groups of CHI immediately form polyelectrolyte complex (PEC) with sulfo and carboxyl groups of Hep, which limits the possibility of mixing and obtaining a homogeneous solution for cryogel synthesis. There is a limited number of studies related to PEC-based cryogel scaffolds, however, extensive research has been done on hydrogel-based PEC, especially with CHI [23]. In one study, researchers were able to obtain CHI-Hep cryogel by blending PEC followed by glutaraldehyde (GA) cross-linking [24]. Previously, the possibility of preparing a biocompatible PEC cryogel composed of CHI, gelatin, and dextran dialdehyde that possesses an internal porosity of cryogel walls was shown [25]. In the current research, we produced CHI-PVA-Hep-GA biocompatible PEC cryogel that also possesses internal porosity of cryogel walls as a sign of PEC formation [25,26]. Figure 1 represents PEC cryogel structure, highlighting the interactions between each component. We hypothesized that introduction of Hep into CHI-PVA cryogel would allow the immobilization of bone morphogenic protein 2 (BMP-2) without additional chemical modifications of a growth factor or cryogel. Obtained cryogel, which was biocompatible with the cells, possessed biological activity and supported differentiation of rat bone marrow mesenchymal stem cells (BMSCs) into osteogenic lineage via release of BMP-2.

## 2. Materials and Methods

### 2.1. Materials

CHI (low viscosity), GA (25% *v*/*v*), and PVA were all from Fisher Scientific, Loughborough, UK. Leucine, Dulbecco’s modified Eagle’s medium—high glucose (DMEM), dexamethasone, β-glycerophosphate disodium salt hydrate 98%, ascorbic acid-2-phosphate, 75 µm HA particles, fetal bovine serum (FBS), glycine, sodium borohydride (NaBH_4_), phosphate buffered saline (PBS) and Rhodamine B were all from Sigma-Aldrich (St. Louis, MO, USA). Hep ≥ 150 IU/mg (ACROS Organics, Fair Lawn, NJ, USA), antibiotic cocktail (penicillin and streptomycin, Gibco, Grand Island, NY, USA), rhBMP-2 (Sigma-Aldrich, St. Louis, MO, USA) and rhBMP-2 ELISA kits (R&D Systems, Minneapolis, MN, USA), Alizarin Red (ACROS Organics, Fair Lawn, NJ, USA), MTT Cell Growth Assay Kit (CT01, Merck, Temecula, CA, USA), mouse monoclonal Osteocalcin antibody (OCG3) (ab13420, Abcam, Cambridge, UK), goat anti-mouse IgG, Superclonal Recombinant Secondary Antibody, Alexa Fluor 488, and lysozyme (Thermo Fisher, Rockford, IL, USA).

### 2.2. Synthesis of Composite Cryogels

CHI of 2% *w*/*v* in 1% *v*/*v* acetic acid was prepared. The powder of HA (20% *w*/*v*) was slowly added into CHI solution (5 mL) and mixed by stirring. A solution of the two remaining ingredients, Hep (2.5 mL with concentration of 1 mg/mL) and PVA (2.5 mL), was prepared. PVA was dissolved in deionized (DI) water (5% *w*/*v*) under prolonged heating at 80 °C for a few hours. Both CHI/HA suspension and Hep/PVA solution were kept on ice. The final mixture was obtained by mixing CHI/HA suspension (5 mL) with Hep/PVA solution (5 mL). The final concentrations of CHI, HA, PVA, and Hep were 1%, 10%, 1.25%, and 0.25 mg/mL, respectively (unless indicated otherwise for the target cryogels). After the addition of GA to the final concentrations of 0.5% (*v*/*v*) and stirring for 10 s, 0.3 mL of the final suspension was quickly transferred into glass tubes (*d* = 7 mm) with silicone plugs at the bottoms. Tubes were placed into a refrigerated circulator (Julabo F34 HE, Seelbach, Germany) at −12 °C and incubated overnight. The following day, the tubes with formed cryogels were removed from the cryostat, plugs were removed, and gels were thawed at room temperature. Then, the cryogels were incubated with 2% glycine in order to block unreacted aldehyde groups in cryogel for 12 h [27] and washed by passing 10 mL of deionised water through each tube. The washed cryogels were freeze-dried until use. Control cryogels with composition of 2% CHI, 10% HA, and 0.5% GA were prepared according to the procedure described above. Non-cross-linked CHI-HA-PVA-Hep cryogels were prepared as described above but without GA.

### 2.3. Swelling Properties of Cryogels and Porosity

Freeze-dried cryogel was sliced into small disc pieces (*n* = 3, *d* = 7 mm, 2 mm thickness), weighed, and placed into DI water. At different time-points (20 s, 40 s, 1 min, 1 min 20 s, 1 min 40 s, 2 min, 1 h) cryogel samples were carefully removed and weighed again. The equilibrium swelling degree was determined by measuring the ratio of the fully swollen cryogel mass (*m*_swollen_, g) to that of its dry mass (*m*_dry_, g):

Swelling degree = (*m*_swollen_ − *m*_dry_)/(*m*_dry_). (1)

Relative porosity of cryogel was estimated by conducting water uptake experiments [28]. Freeze-dried cryogel cylinders were incubated in DI water until reaching equilibrium. The weight of swollen cryogels (*m*_swollen_, g) were measured, and after that the swollen cryogels were squeezed to remove excess water and the mass (*m*_squezzed_, g) was recorded. Estimated porosity was calculated according to the following equation:

Porosity (%) = ((*m*_swollen_ − *m*_squezzed_)/(*m*_swollen_)) × 100. (2)

### 2.4. Mechanical Testing

The compressive moduli of the cryogel columns were determined at room temperature on the Texture Analyser TAXT Plus (Stable Micro System, Surrey, UK) under unconfined uniaxial compression with a 5N (five-Newton) load cell. Prior to making any measurements and testing for mechanical properties, cryogels were incubated with DI water until they reached swelling equilibria. 5N load was used to compress cryogels at the rate of 0.05 mm/s. The compressive Young’s modulus was calculated from the initial linear portion of the curve (<10% strain) as a mean value from at least five samples using the following equation:

*E* = (*F*/*S*)/(Δ*h*/*h*) (3)

where *E* is the elastic modulus (Pa), *F* is the force applied (N), and *S* is the cross-sectional area of the sample (m^2^, Δ*h* is the height (m) at compression, and *h* is the original height (m)) [13].

### 2.5. In Vitro Degradation

Prior to taking any measurements, cryogels were sterilized by incubation in 70% ethanol for 2 h, washed with DI water, and freeze-dried. The degree of degradation α (%) was calculated by comparing initial freeze-dried weight of cryogels (*m*_i_—initial mass in mg) to freeze-dried weights at different time points after incubation in PBS at 37 °C for 1, 2, and 4 weeks. At each time point, cryogels were washed with DI water, freeze-dried, and weighed (*m*_f_—final mass in mg). α (%) was calculated according to the following equation:

α (%) = ((*m*_i_ − *m*_f_)/*m*_i_)) × 100. (4)

Additionally, cryogels were incubated with PBS-containing lysozyme (10,000 U/mL) following the same protocol as described above.

### 2.6. Rheological Analysis

Flow behavior and viscoelastic properties of the solutions were measured using a HAAKE RheoStress 1 Rheometer (Thermo Scientific, Stone, UK) with a cone and plate geometry. The gap between the cone and plate was 0.5 mm. In order to determine the linear viscoelastic region of the tested gel solutions, amplitude sweep tests were performed at a constant frequency (ω) of 10^−1^ and an amplitude range of 0.1–100%. All experiments were performed at 25 ± 1 °C.

### 2.7. FTIR

In order to characterize the cross-linking polymers and Hep attachment, infrared measurements were carried out with a Universal ATI FTIR spectrometer (Perkin Elmer, Spectrum 650, Waltham, MA, USA). Freeze-dried cryogels were crushed using a mortar and pestle and were then analyzed with FTIR spectroscopy. FTIR spectra were obtained in the range of 4000–650 cm^−1^ during 64 scans, with 2 cm^−1^ resolution, using diffuse reflectance mode.

### 2.8. Microscopy

The porosity and internal structure of the cryogels were visualized using optical microscopy (EVOS FL Auto 2 Cell Imaging System, Bothell, WA, USA), confocal laser scanning microscopy (Carl Zeiss, LSM 780, Jena, Germany), and scanning electron microscopy (Jeol, JSM-IT200 InTouchScope, Tokyo, Japan). For confocal imaging, cryogel slices (1 mm thick) were incubated with 1 mL of 50 mM Rhodamine B for 30 min in the dark followed by an extensive wash with DI water. Excitation and emission wavelengths were chosen according to the manufacturer’s instructions. For SEM imaging, freeze-dried cryogel slices without coating were used. Pictures were taken at variable accelerating voltage and magnifications.

### 2.9. Release of BMP-2 from Cryogel Matrix

Cryogel slices (*n* = 3, *d* = 7 mm, 2 mm thickness) were cut into four small pieces and incubated with 20 ng/mL rh-BMP-2 overnight at room temperature on a shaker at 50 rpm. Afterwards, the cryogels were carefully removed and transferred to the tube with fresh 1 mL PBS. Supernatants were collected at different time points (days 1, 7, 15, and 30), with fresh PBS added each time. Collected supernatants were analyzed for BMP-2 loading efficiency and release kinetics using ELISA assay according to the manufacturer’s instructions.

### 2.10. MTT Assay

Sterile freeze-dried CHI-PVA-HA-Hep-GA cryogel slices (*n* = 3, *d* = 7 mm, 2 mm thickness) previously Schiff's base reduced with 50 mM NaBH_4_ were grinded into a powder and incubated in 1 mL growth media (10% FBS, DMEM, 1% penicillin/streptomycin) for 6 h. After incubation, the supernatants were collected by centrifugation at 6000× *g* for 10 min and sterile filtered through a 0.22 µm syringe filter. NIH/3T3 cells at a concentration 5 × 10^4^ were seeded into a 96-well plate and incubated at 37 °C in a humidified 5% CO^2^ atmosphere for approximately 18 h prior to treatment with collected cryogel supernatants. A total of 10 µL of supernatant was mixed with 90 μL of fresh media and added into each well with the NIH/3T3 cells. A negative control containing only culture media was also included. The cells were further incubated with samples for 24, 48, and 72 h. Afterwards, absorbance at 570 nm was assessed using an MTT Cell Growth Assay Kit according to the manufacturer’s instructions.

### 2.11. In Vitro Effect of Scaffolds Containing rhBMP-2 on Rat BMSC Mineralization

Cryogel slices, (*n* = 5, *d* = 7 mm, thickness = 2 mm), which were inactivated with 50 mM NaBH_4_, were cut into four pieces and incubated overnight in 1 mL of BMP-2 solution (1 µg/mL). Rat BMSC, passage 5, was used to evaluate the effect of BMP-2 released by cryogel. BMSC cells were seeded (1 × 10^5^ cells per well in a 24-well plate) in osteogenic medium that was replaced every three days. Osteogenic medium was prepared and composed of DMEM, FBS 10%, 50 μg/mL ascorbic acid, 10 mM β-glycerophosphate, and 10 nM dexamethasone. In order to assess the effect of BMP-2 released from cryogels, cells were divided into three groups: cultured in osteogenic media without cryogel (OS media), cultured in osteogenic media with cryogel pre-loaded with BMP-2 (OS-BMP media), and growth media only (DMEM, 10% FBS, 1% penicillin/streptomycin). At each time point (7, 14, and 21 days), cells were fixed with 2.5% GA for 30 min and washed three times with PBS before being stained with 1% Alizarin Red solution (pH 4.2) for 10 min. Afterwards, Alizarin Red solution was aspirated and the cells were washed with PBS and air-dried before taking color images (EVOS FL Auto 2 Cell Imaging System, Thermo Fisher Scientific, Bothell, WA, USA).

### 2.12. Rat BMSC Culture on Cryogel

Cryogel slices (*n* = 5, *d* = 7 mm, thickness = 2 mm), which were neutralized with 50 mM NaBH_4_, were incubated overnight in 1 mL of BMP-2 solution (1 µg/mL). Rat BMSC passage 5 was seeded (1 × 10^5^ cells per well in a 24-well plate) on top of cryogels and supplied with fresh osteogenic media every 3 days for 2 weeks. After 14 days, the cryogels were fixed with 2.5% GA overnight at +4 °C and washed three times for 10 min with PBS. Afterwards, they were stained with mouse monoclonal osteocalcin antibody (OCG3) and then with goat anti-Mouse IgG (H+L), Superclonal recombinant secondary antibody, and Alexa Fluor 488 according to the manufacturer’s instructions. The cryogels were washed with PBS and stained with DAPI for 5 min and washed with PBS three times. Stained cryogels were visualized on a ZEISS LSM 880 confocal laser scanning microscope.

### 2.13. Statistical Analysis

Data is represented as mean ± SD and all analysis was done using an unpaired *t*-test. For experiments with more than three groups per time points, one-way ANOVA was used. *p*-value < 0.05 was considered statistically significant. All experiments were carried out at least in triplicate (*n* = 3) unless otherwise specified.

## 3. Results

### 3.1. Cryogel Synthesis and Physico-Chemical Characterization

**Figure 2:**
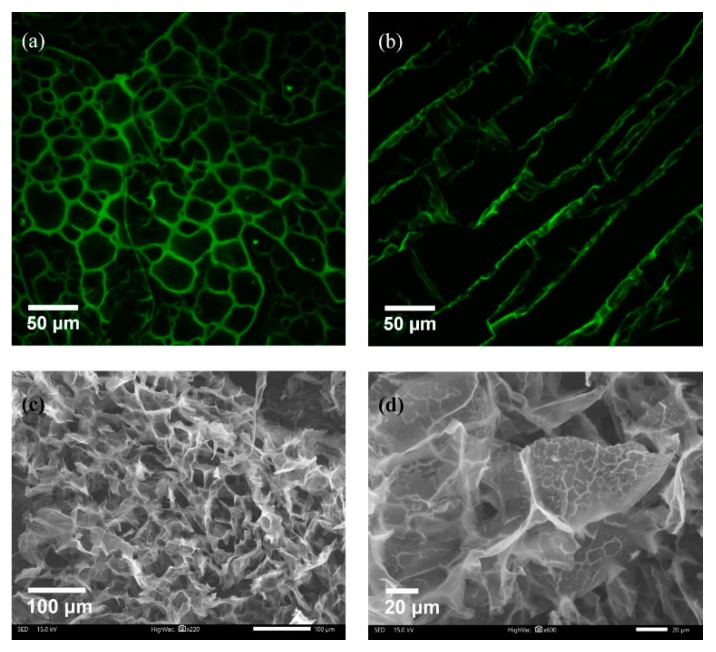
Porosity of CHI-PVA-Hep-GA cryogel. Confocal laser scanning cross-sectional (**a**) and longitudinal images (**b**) of Rhodamine B-stained cryogels showing the porous structure with interconnected pores. SEM imaging confirming porosity (**c**) and presenting internal porosity of the cryogel walls (**d**) as a sign of polyelectrolyte complex (PEC) formation.

A composite CHI-PVA-HA-Hep-GA cross-linked cryogel was prepared. Additionally, a series of control cryogels without Hep—CHI-PVA-HA-GA, CHI-GA, and CHI-HA-GA—were synthesized and used for estimation of the effect of each component on the mechanical properties of obtained cryogel. Monolithic columns of composite macroporous cryogel were synthesized via a polycondensation reaction of CHI premixed with HA and PVA premixed with Hep and GA as a cross-linking agent at −12 °C. Synthesized composite cryogels had large continuous interconnected pores (Figure 2a–d) that acted as channels, which along with the available surface area, permitted intermolecular binding and passing through of substances.

The swelling ratio of CHI-PVA-HA-Hep-GA was around 9.8 ± 0.14 after 1 h incubation (data not shown). SEM images (Figure 2c,d) confirmed the porous structure of obtained cryogel and PEC structure presence indicated by the porosity of the cryogel walls (Figure 2d).

### 3.2. Mechanical and Rheological Properties

**Figure 3:**
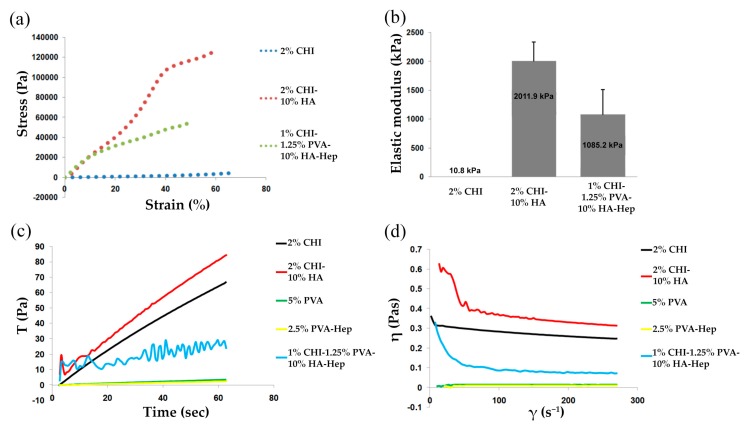
Rheological and mechanical properties of cryogels. Stress–strain curves (**a**) and elastic moduli (**b**) for each cryogel group. Viscosity at constant shear rate (**c**) and viscosity versus shear rate plot (**d**).

Figure 3a shows representative stress–strain curves for each cryogel group, where all cryogels displayed a concave upward curve characteristic of elastomeric materials with large deformation. CHI-HA-GA cryogels had a significantly larger elastic modulus (2011.9 ± 328.80 kPa) in comparison to CHI-GA and CHI-PVA-HA-Hep-GA (10.8 ± 0.47 kPa and 1085.2 ± 427.97 kPa, respectively) (Figure 3b). The increase of the elastic modulus could be explained by the presence of HA, which increased the mechanical properties of the cryogels.

The flow behavior of CHI-PVA-HA-Hep blends lay between those of the pristine polymers (CHI and PVA), and the results of the time-dependent viscosity measurements of the different blend solutions are shown in Figure 3c,d. The viscosity of CHI did not significantly change upon applied shear stress range, and the viscosity of the CHI-PVA-HA-Hep solutions was higher than that predicted by the rule of mixtures [29]. The detailed mechanism of interactions between polymers using rheology was out of the scope of the current study. At high shear rates, the decrease in viscosity could be caused by the applied shear. With a high level of applied stress on CHI-PVA-HA-Hep, the network became more compact due to additional mixing, which triggered the formation of more stochiometric PEC between the oppositely charged groups of polymers, which was more hydrophobic and had a tendency to collapse due to hydrophobic interactions. Therefore, a viscosity drop in the blend solution could be caused by the stress-induced additional interaction of available functional groups of CHI-PVA-HA-Hep. The highest viscosity was observed in CHI-HA blend suspension and the lowest in PVA and PVA-Hep solution due to its low molecular weight. Furthermore, characteristic multiple inflections were observed in the shear rate curve of CHI-PVA-HA-Hep blend (Figure 3c). The presence of these inflections could be the result of the creation and tearing of local structures. In this case, PEC between CHI and Hep possessed a more compact structure and was more hydrophobic, which led to collapse and aggregation under applied stress.

### 3.3. FTIR

**Figure 4:**
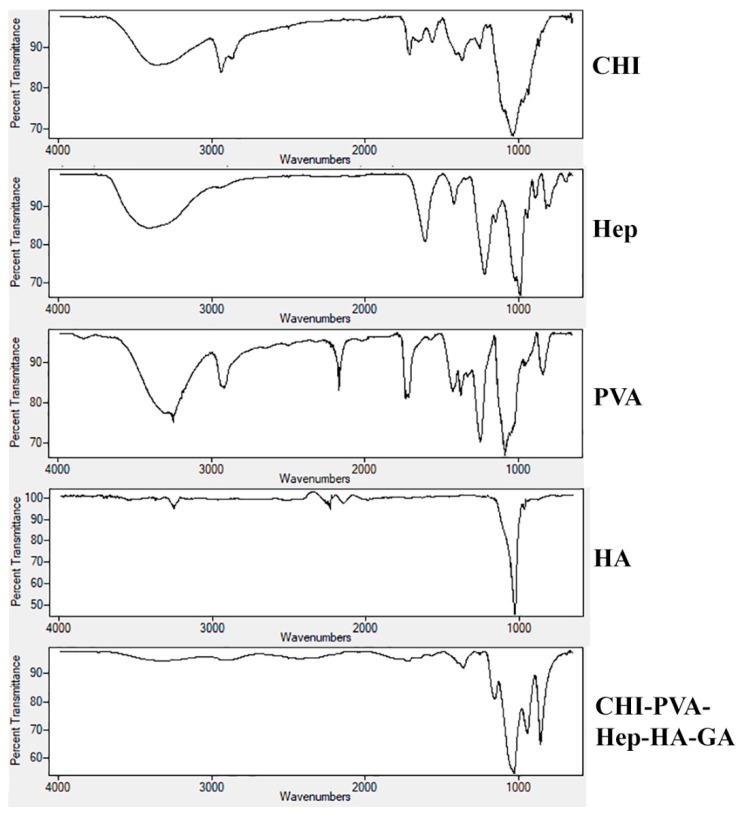
FTIR spectra of individual components CHI, PVA, Hep, HA, and the composite cryogel. Frequencies of functional groups of CHI, PVA, and Hep were detected at 2929–2880 cm^−1^ (–C–H), 1548 cm^−1^ (–NH_2_), 1404 cm^−1^ (the coupling of –C–N and –N–H), 1062 and 1026 cm^−1^ (–C–O), and 896 cm^−1^ (C–C ring of CHI and heparin).

A comparative analysis of FTIR spectra of initial components of CHI, PVA, Hep, HA, and the composite cryogel is presented in Figure 4. The carbonyl stretching frequency of the amide bond of CHI was confirmed by the peak at 1635 cm^−1^ [10]. The reaction between the primary amino groups of CHI and aldehyde of GA led to a shift in amino bond frequency from 1550 to 1590 cm^−1^. A wide peak at 3270–3400 cm^−1^ attributed to hydroxyl and the primary amine groups of CHI [10], PVA, and Hep. Frequencies of functional groups of CHI, PVA, and Hep were detected at 2929–2880 cm^−1^ (–C–H), 1548 cm^−1^ (–NH_2_), 1404 cm^−1^ (the coupling of –C–N and –N–H) [10], 1062 and 1026 cm^−1^ (–C–O), and 896 cm^−1^ (C–C ring of CHI and Hep). For the chemically cross-linked composite cryogels CHI-PVA-HA-Hep and CHI-PVA-HA, a frequency shift from 1633 to 1592 cm^−1^ was related to Schiff’s base formation. In the case of the cryogel CHI-PVA-HA-Hep, one can observe that all characteristic frequencies of the functional groups of polymers shifted, indicating the interactions between functional components of polymers, such as electrostatic interactions. Due to high concentration of HA in the cryogel composition compared to polymers, the intensity of their characteristic groups was significantly lower compared to FTIR peaks of the initial polymers. A small intensity peak appeared at 820 cm^−1^, which was associated with C–S–O group of Hep in the structure of the cryogel. The absorbance at 1230 cm^−1^ was attributed to asymmetric S = O bond [30].

### 3.4. In Vitro Cryogel Degradation and BMP-2 Release Rate

*In vitro* degradation in PBS for a period of 4 weeks (Table 1) revealed that the composite CHI-PVA-HA-Hep-GA cryogel degraded slowly and was more stable in comparison to CHI-GA and CHI-HA-GA cryogels, which could be due to PEC formation between CHI-Hep in addition to GA cross-linking. CHI-based cryogels cross-linked by dextran dialdehyde and PEC-based cryogel composed of CHI and gelatin illustrated a degradation degree in the range of 10% to 15%, whereas non-reduced Schiff’s base cryogels degraded in the range of 25% to 35% [25].

**Figure 5:**
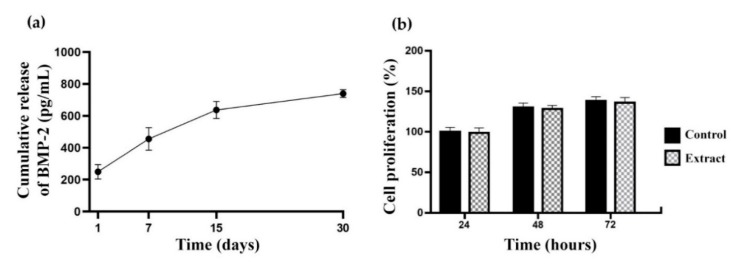
Cumulative release of bone morphogenic protein 2 (BMP-2) from the composite cryogel over 30 days (**a**) and MTT assay results (**b**) showing no negative effect of CHI-PVA-HA-Hep-GA cryogel extract on proliferation of NIH/3T3 cells. Values represent mean and standard deviation (*n* = 3).

ELISA results in Figure 5a show cumulative release of BMP-2 from CHI-PVA-HA-Hep-GA cryogels for the total of 30 days incubation. BMP-2 loading efficiency was approximately ~83.2%. Cumulative release of BMP-2 from cryogel after 30-day incubation in PBS was 700 pg/mL corresponding approximately to 4.52% of the bound BMP-2. These data confirm affinity between Hep and BMP-2, and its release could be connected with the degradation of cryogel matrix. Figure 5b shows no cytotoxic effect of CHI-PVA-HA-Hep-GA cryogel extract on NIH/3T3 cells after 24, 48, and 72 h incubation.

### 3.5. Effect of Cryogels Loaded with BMP-2 on Rat BMSC Mineralization

**Figure 6:**
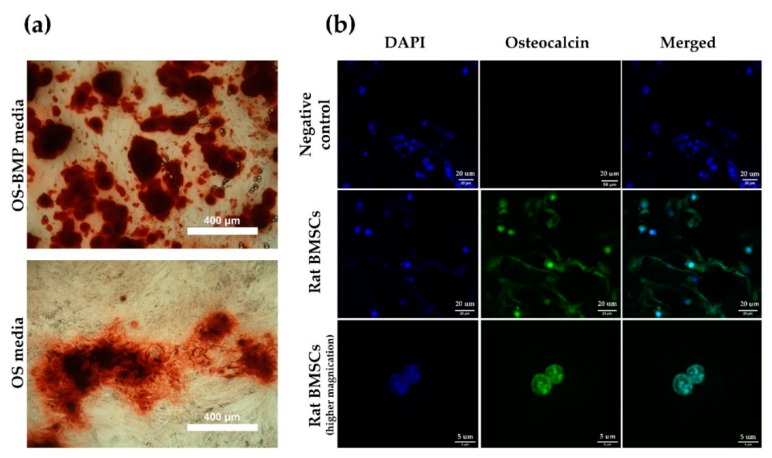
Osteogenic (OS) differentiation. Mineral nodule formation after Alizarin Red S staining on day 21 (**a**) and CLSM images of rat BMSCs growing on CHI-PVA-Hep-GA cryogels (**b**). Rat BMSCs were grown on cryogel in osteogenic media and stained with DAPI (blue) and anti-osteocalcin antibodies (green). Negative control—rat BMSCS grown on cryogel in Dulbecco’s modified Eagle’s medium (DMEM), 10% fetal bovine serum (FBS).

Bone mineralization resulting from calcium deposition is known as a late marker in osteogenic differentiation. Most mineralization was initiated after 14 days. Rat BMSCs incubated in OS-BMP media (Figure 6a) mineralized by day 21, as visualized by the darker color, rougher particles, and widely branched deposits, in comparison to the cells incubated in OS media only. Overall, the mineralization was not efficient, it could be explained by the use of primary and freshly isolated rat BMSCs, which were heterogeneous and not enriched further against MSC markers. Moreover, rat BMSCs grown for 2 weeks on BMP-2 loaded CHI-PVA-Hep-HA-GA cryogel (Figure 6b) expressed osteocalcin and confirmed the biocompatibility of the obtained cryogel without any cytotoxic effect and interference to osteogenic differentiation.

## 4. Discussion

The extracellular matrix plays an essential role in tissue integrity and regeneration, and mimicking this matrix is important for designing bioscaffolds applicable for tissue engineering purposes. Owing to their macroporous structure, biocompatibility, and good mechanical properties, cryogels have become a popular material for tissue engineering purposes [31,32]. Moreover, due to their unique sponge-like structure, cryogels have been used to create scaffolds, bioreactor systems, and many other materials [11,33,34]. For example, cryogels improve the production of antibodies and the process of cryopreservation [25,32,35,36]. As a bioreactor, they have been used for cell culture and cell separation [37,38]. However, more importantly, they have proven to be good candidates for bioscaffold engineering, showing potential in tissue regeneration applications [12,39].

The main aim of the current research was to develop a suitable cryogel scaffold that possessed the ability to promote bone regeneration via suitable mechanical properties for implantation, as well as the ability to bind BMP-2 and to be tissue compatible and biodegradable. CHI, PVA, and Hep were chosen in order to obtain cryogels that will enable selective and strong adsorption of BMP-2 without additional modification. Release kinetics and degradation experiments have shown efficient loading of BMP-2 (~82.3%) and cryogel degradation-dependent release of bounded growth factor. As shown in Table 1, CHI-PVA-HA-Hep-GA cryogel lost 7% of its weight, which correlated with the 4.52% (700 pg) release of BMP-2. Heparin-binding sites are present in BMP-2 [40] and not only affect its affinity, but also its biological activity [41]. It was also shown that HA has natural properties to absorb BMP-2, which is done via −OH, −NH_2_, and −COO− functional groups of BMP-2 [42]. Therefore, to reinforce the strength of the cryogel for compression or other types of stress and to increase biocompatibility and accelerate biomineralization of the cryogel, HA was introduced. Mechanical measurements showed that addition of HA significantly increased the elastic modulus of CHI-PVA-HA-Hep-GA cryogel (1085 ± 428 kPa) and that the value was significantly higher in comparison to other cryogel-based scaffolds designed for bone regeneration [43,44,45,46,47,48,49]. Degradation experiments confirmed a decreased in vitro degradation rate of HA-containing cryogels, which is in line with other published data [45,50]. A lower degradation rate could be explained by the reduction of Schiff’s base groups after NaBH_4_ addition, which removes hydrolytically active groups [51]. In vivo CHI is degraded by lysozyme, and according to the published data, could lose more than 50% of its weight within 30 days [52,53], and our data supports this. Confocal and SEM images of composite cryogels showed high porosity and interconnective pore morphology, which were very suitable structural properties for porous materials in tissue engineering. CHI is a positively charged polymer with the ability to form PEC with Hep, and it is impossible to prepare a CHI-Hep cryogel due to the instant formation of a hydrogel. Through experimental design, it was found that pre-mixing of Hep with PVA inhibited immediate formation of PEC after addition of CHI, possibly due to the formation of a non-stoichiometric interpolymer complex (IPC) between Hep and PVA at chilled conditions. Decreased temperature facilitated the formation of hydrogen bonds and therefore IPC. Rheological experiments have shown fluctuations in the flow curve of CHI-PVA-Hep-HA mixture. The formation of PEC between CHI and Hep in the presence of PVA and HA was confirmed by a significant decrease in viscosity of the solution compared to initial components such as CHI and the combination of CHI-HA, and inflections were visible on the shear rate curves. These changes were not associated with the addition of HA, as shear rate of CHI-HA is normal. From these data, we could conclude that fluctuations are the result of the creation and tearing of local structures, or PEC between CHI and Hep. The detailed mechanism of interactions between polymers is out of scope of the current study. Previously, the formation of IPC between PVA and negatively charged polymers such as polysaccharide, polyaspartic acids, and polyacrylic acid were reported [54,55,56]. Formed IPC associated with PVA-Hep led to a hindrance of sulfo groups of Hep with CHI, slowing down the formation of PEC at the studied temperature and concentration. The freezing of the mixture of PVA-Hep and CHI-HA led to the formation of nonstochiometric PEC between oppositely charged groups of CHI and Hep due to the cryoconcentration effect. Moreover, SEM images showed the formation of pores within cryogel walls, which is in line with our previously published data where we showed internal pore formation on the cryogel walls due to PEC [25].

An important property of these cryogel scaffolds is their swelling ability and behavior. Composite cryogels in aqueous media swelled within a few minutes, and reached a swelling equilibrium in 20–25 min. The average swelling ratio of CHI-PVA-HA-Hep-GA cryogels was 9.5 ± 0.84. The average elastic moduli for the cryogel CHI-PVA-HA-Hep-GA could reach 1085.2 ± 427.9 kPa, which is associated with the presence of a high concentration of HA. Also, 10% HA by *w*/*w* increased the toughness of cryogel, which made it a suitable substrate for implantation into the bone. CLSM showed that rat BMSCs attach and grow on cryogels loaded with BMP-2, without showing a cytotoxic effect from the scaffold, and start to express osteocalcin. Hep within cryogel allows the loading of growth factors such as BMP-2 via electrostatic interactions, which was used in this study. Additionally, BMP-2 has a Hep-binding domain [57]. *In vitro* experiments have shown that obtained cryogels release BMP-2 and support differentiation of rat BMSCs into osteogenic lineage and that its release is associated with the physical biodegradation of the cryogel. With some modification, the cryogels could be successfully used as tissue-engineered scaffolds. Incorporation of various additives can be done to adjust both the structure and function of the scaffold to mimic the tissue environment for tissue engineering applications.

## 5. Conclusions

In this study, we evaluated the possibility of a one-step preparation of a scaffold for growth factor delivery, such as BMP-2, without additional chemical modifications using a cryogelation technique. Our studies have demonstrated that PEC cryogels obtained from natural ingredients, such as Hep and CHI, are appropriate vehicles for cell proliferation and differentiation. In this work, we demonstrated the potential application of the CHI-Hep-based PEC cryogels as a carrier of growth factors for rat BMSCs differentiation. The growth factors could be loaded into cryogel in a one-step manner without any chemical modifications and without losing their biological activity. Current research has shown the potential use of cryogel matrix made from natural components, such as CHI and Hep, for a growing number of tissue engineering applications. In our future work, we plan to demonstrate the clinical efficacy of prepared cryogel for bone regeneration in an animal model.

## 6. Patents

Patent application for the preparation of CHI-PVA-Hep-GA has been submitted in the Republic of Kazakhstan.

## Figures and Tables

**Table 1:** Degradation of composite cryogels in PBS and PBS-lysozyme solution for a period of 4 weeks.

Sample	Degradation (%)			Degradation (%) in Lysozyme (10,000 U/mL)		
	1 Week	2 Weeks	4 Weeks	1 Week	2 Weeks	4 Weeks
CHI-GA	5.50 ± 0.71	11.93 ± 0.39	20.43 ± 0.18	19.48 ± 0.62	25.54 ± 0.40	41.22 ± 0.60
CHI-HA-GA	2.50 ± 0.70	4.68 ± 0.62	8.08 ± 0.41	8.65 ± 0.31	14.70 ± 0.35	22.55 ± 0.55
CHI-PVA-HA-Hep-GA	2.58 ± 0.60	5.20 ± 1.32	6.99 ± 0.16	10.22 ± 0.30	18.90 ± 0.95	25.61 ± 0.70
